# Definition and classification of ‘travellers’ in research: a bibliometric analysis

**DOI:** 10.1093/jtm/taae048

**Published:** 2024-03-19

**Authors:** Wondimeneh Shiferaw, Judith A Dean, Deborah Mills, Colleen Lau, Luis Furuya-Kanamori

**Affiliations:** UQ Centre for Clinical Research, Faculty of Medicine, The University of Queensland, Herston, QLD 4006, Australia; Asrat Woldeyes Health Science Campus, Debre Berhan University, Debre Berhan, Ethiopia; School of Public Health, Faculty of Medicine, The University of Queensland, Herston, QLD 4006, Australia; UQ Centre for Clinical Research, Faculty of Medicine, The University of Queensland, Herston, QLD 4006, Australia; Dr Deb The Travel Doctor, Travel Medicine Alliance, 445 Brisbane, QLD 4006, Australia; UQ Centre for Clinical Research, Faculty of Medicine, The University of Queensland, Herston, QLD 4006, Australia; UQ Centre for Clinical Research, Faculty of Medicine, The University of Queensland, Herston, QLD 4006, Australia

**Keywords:** Travel, travellers, bibliometric, classifications, definition, tourism, international travel

## Abstract

Despite the growth in the volume of vulnerable travellers globally, there has been limited research attention, underscoring the need for more evidence as these individuals are susceptible to various travel-related health risks. Additionally, we observed inconsistencies in the definition of travellers and discrepancies in travel-related publications compared to the actual travel volume.

The 2022 United Nations World Tourism Organization (UNWTO) report revealed that international travellers surpassed 960 million trips annually, and it is estimated to reach nearly 2 billion trips by 2030.^1^ As population mobility expands to diverse regions, it presents substantial challenges for designing public health programs and policies to control disease importation and safeguard the health of travellers and local populations. Travel medicine has emerged as a growing discipline over the past three decades,^2^ driven by the increasing global mobility and growing tourism industry. Travel medicine practice and guidelines rely on robust research to inform an evidence-based decision-making approach. However, ‘travellers’ are a heterogeneous group in terms of their demographics, risk perception, uptake of pre-travel healthcare interventions, health status (e.g. comorbidities) and travel itineraries.^3^ This variability limits the generalisability of research findings, and therefore their application and relevance in clinical practice.

Classifying the number of research studies by types of travellers (e.g. tourist, business, and visiting friends and relatives [VFRs]) holds significant advantages for exploring gaps in evidence and allowing for better understanding of specific travellers’ needs. Specific evidence for each type of traveller would enable the implementation of tailored preventive and treatment measures, enhancing the travel experience, and reducing the risk of morbidity and mortality.^4^ Moreover, it would facilitate the development of risk assessment tools and policies that consider the unique risks associated with each type of traveller. Previous bibliometric analyses conducted of the *Journal of Travel Medicine* (JTM)^5^ and *Travel Medicine and Infectious Disease* (TMAID),^6^ primarily focused on travel medicine topics and areas of research. However, in this bibliometric analysis study, we aimed to quantify travel medicine research by type of travellers and contrast the amount of research with the global volume of each type of traveller.

Firstly, given the large heterogeneity in travellers, we reviewed the UNWTO, Centers for Disease Control and Prevention Yellow Book, World Health organization International Travel Health, International Organization for Migration and United Nations Department of Economic and Social Affairs guidelines and policy documents to identify the definition(s) of ‘traveller’ used by these key organizations. Secondly, we undertook a bibliometric analysis of all articles published in the JTM and TMAID over the past 5.5 years (i.e. January 2018 to July 2023) to determine the number of publications by types of travellers. We selected these journals due to their specific focus on travel medicine, visibility and reputation in the field. Articles from these journals were retrieved and imported into EndNote X9 (Clarivate Analytics, Philadelphia, PA). WS conducted the search, retrieved and screened the articles for inclusion. LFK verified the screening process by reviewing 10% of the retrieved articles. Travel-related publications involving humans that presented new data/evidence were included. Publications that did not present new data or were based on opinions (e.g. commentaries) and COVID-19 papers were excluded. Thirdly, we extracted annual travel volume (by type of traveller) from the UNWTO for the past 5 years (i.e. 2018 to 2023).^1^ However, we were unable to obtain travel volume data by type of travellers for 2020/21. This might be due to the impact of the COVID-19 pandemic on international travel restrictions. We compared the proportion of travel volume and research articles by type of travellers (grouped based on their planned travel-related activities) using the Chi-square (*χ*^2^) statistic.

We identified definitions for 27 different types of travellers from international guidelines and policy documents and grouped them into three categories based on (i) planned travel-related activities (e.g. tourists, VFRs, business travellers), (ii) travellers’ baseline characteristics (e.g. vulnerable travellers with medical conditions) and (iii) duration of trip (e.g. long-term travellers) (Supplementary Material Material S1). In the bibliometric analysis of journal publications, 2168 articles were retrieved, of which 522 met the inclusion criteria (Supplementary Material Material S2). It is important to note that out of the 522 articles included, several studies (*n* = 102) reported multiple types of travellers in a single study, and the number of articles increased to 851 when each type of traveller within those studies was considered individually. Research was conducted in 20 distinct types of travellers; most research articles defined the type of travellers based on the planned travel-related activities (*n* = 862, 91.6%), followed by travellers’ baseline characteristics (*n* = 68, 7.2%). A small number of publications defined the type of traveller based on the duration of the trip (*n* = 11, 1.2%). The number (and proportion) of publications by specific type of travellers is presented in Table 1.

**Table 1:** The proportion of research articles published in the *Journal of Travel Medicine* and *Travel Medicine and Infectious Disease*, by type of travellers, from 2018 to 2023

Type of travellers	Number of articles (%)
Travellers baseline characteristics	
Vulnerable travellers	59 (11.3)
Immunocompromised travellers	22 (4.2)
Paediatric travellers	12 (2.3)
Travellers with underlying disease	9 (1.7)
Older travellers	8 (1.5)
Pregnant travellers	6 (1.1)
Obese travellers	2 (0.4)
LGBTQIA+ travellers (MSM and LGTB travellers)	9 (1.7)
Planned travel-related activities	
Business travellers	132 (15.3)
Crew members (ship and aircrew)	25 (2.9)
Expatriate	27 (3.1)
Business	80 (9.3)
Tourists	214 (24.8)
Adventure (trekker/hikking, safaris, ayahuasca)	57 (6.6)
Backpackers	11 (1.3)
Sex tourist	4 (0.5)
Tourist (tourism, leisure/recreation, visitors, holiday, space and festival)	142 (16.5)
VFRs, health and religion	144 (16.7)
Medical/health tourism	17 (2.0)
Religion (e.g. Hajj, Umrah and Pilgrim’s travellers)	42 (5.0)
VFRs	85 (9.7)
Others	291 (33.8)
Students (medical student abroad, research, gay year, humanitarian aid)	54 (6.3)
Forcibly displaced persons (refugees, asylum seekers and displaced people)	48 (5.5)
Immigrants	24 (2.8)
Migrant (seasonal migrant worker, short-term migrant and migrants)	92 (10.7)
Humanitarian/volunteers travellers	46 (5.3)
Military	27 (3.1)
Unspecified (traveller, returned, international and last minute)	81 (9.4)
Duration of trip	
Long-term travellers (i.e. relocation, diaspora and adoption)	11 (2.1)
Total	851 (100)

LGBTQIA+: lesbian, gay, bisexual, transgender, queer or questioning, intersex, asexual and more; MSM: men who have sex with men; LGTB: lesbian, gay, bisexual and transgender.

We identified differences between the proportion of publications conducted by type of travellers and the annual travel volume (*χ*^2^ (3) =126.7; *P* value < 0.001) (Fig. 1). Although tourists constituted the highest volume of travellers (54.5%), a relatively low number of research studies was conducted in this group (24.8%). Conversely, while business travellers accounted for a smaller volume of annual travel (12.3%), a relatively higher proportion of publications (15.3%) was associated with this group. Despite the absolute proportions being quite different, the proportion of research publications reporting on research involving VFRs and travellers for health and religious purposes (16.7%) aligns consistently with the travel volume (29.2%) for these groups. Our findings also highlight a notable gap in research conducted involving vulnerable travellers (e.g. immunocompromised, pregnant, paediatric and obese travellers), despite their higher risk of morbidity and mortality. It is worth noting that ‘Other’ type of travellers in Fig. 1(A) was not included in the comparison between research (Fig. 1B) and travel volume (Fig. 1B), due to the absence of comparable data in the latter.

**Figure 1:**
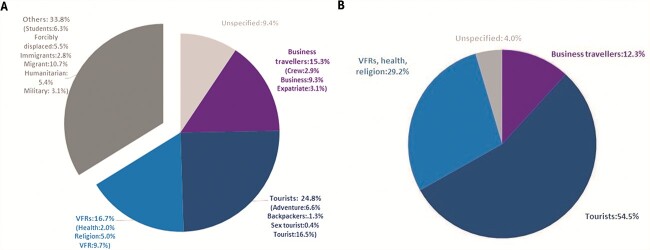
Proportion of (A) research articles published in JTM and TMAID from 2018 to 2023 and (B) annual travellers’ volume for the last 5 years from 2018 to 2023 extracted from the UNWTO by the type of traveller.

Our study has certain limitations. Firstly, the use of publication as a proxy for research may not capture all conducted studies as not all travel medicine related research is published in these two journals. Secondly, during the COVID-19 pandemic, research focus shifted (including travel medicine research priorities) which could have impacted the type of research conducted and the articles published. Thirdly, we combined research articles published in JTM and TMAID. There may be differences in editorial policies and scope between these journals.

In the current study, we observed variation in the definition of travellers and identified a discrepancy between the number of publications and volume of travellers. With the growing number of international travellers, it is feasible to posit that there will be increasing numbers of travellers with underlying medical conditions (i.e. vulnerable travellers). Vulnerable travellers (e.g. older, paediatric, immunocompromised, pregnant and obese travellers) have higher risk and more severe travel-related infections compared to healthy travellers^7^; however, our study highlights that limited research has been conducted in this group of travellers. There is a clear need for more granular evidence to guide healthcare interventions for these populations, and addressing this research gap is essential. Having evidence by type of travellers can provide valuable support to healthcare providers to understand, prevent and respond to the various health issues that different travellers may encounter.

## Funding

The authors did not receive funds for conducting the review. Wondimeneh Shiferaw was granted the University of Queensland Research Training Stipend Scholarship to pursue his PhD.

## Author contributions

Wondimeneh Shiferaw (Conceptualization, Methodology, Investigation (screened articles and extracted data), Formal Analysis, Visualization, and Writing—original draft and editing), Judith A. Dean (Supervision, Writing—review & editing), Deborah Mills (Supervision, Writing—review & editing), Colleen Lau (Supervision, Writing—review & editing), Luis Furuya-Kanamori (Conceptualization, Supervision, Writing—review & editing).

##  

**Conflict of interest**: The authors have declared no conflicts of interest.

## Supplementary Material

Supplementary_material_taae048
