# Supplementary material for: Definition and classification of ‘travellers’ in research: a bibliometric analysis
Source: J Travel Med. 2024 Mar 19;31(4):taae048. doi: 10.1093/jtm/taae048 (PMC11149717; doi:10.1093/jtm/taae048)

**Supplementary material**

**S1.** Definitions of different types of travellers.

| **Definition of travellers** | | **Sources** |
| --- | --- | --- |
| **Travellers baseline characteristics** | | |
|  | **Immunocompromised travellers:** Someone with a suppressed immune status, whether due to a health condition, medication, or other treatment. | CDC yellow book^8^ |
|  | **LGBTQ+ travellers:** Are often exhibit more frequent travel, higher-than-average spending patterns, and a heightened level of brand awareness and loyal. | CDC yellow book^8^ |
| **Planned travel-related activities** | | |
|  | **Adventure traveller**: A person seeking exciting and thrilling experiences in outdoor or adventurous activities. Adventure activities often occur in natural environments, including hiking, rock climbing, white-water rafting, zip-lining, bungee jumping, wildlife safaris, and snowboarding. Due to the nature of the activity the travellers engaged in, they are at higher risk of illness and injury. | CDC yellow book^8^ and UNWTO^9^ |
|  | **Asylum seeker: Someone whose claim has yet to be decided on by the country where they have submitted it. Only some asylum seekers will ultimately be recognized as refugees, but every recognized refugee is initially an asylum seeker who is seeking international protection.** | IOM^10^ |
|  | **Backpackers:**These are young travellers who do not have a fixed itinerary and are on a restricted budget, want to experience adventure and excitement, tend to travel independently, enjoy meeting another traveller, and travel for more extended periods. | Literature |
|  | **Business traveller**: An individual travelling to work (including a range of occupational-related travel, corporate travel, and fieldwork) or attending a meeting or other work-related events such as conferences or research activity. | CDC yellow book^8^ and UNWTO^9^ |
|  | **Crew (air and ship) travellers**: These are a specific category of leisure travellers who are also staff or crew members working on aircraft or ships. These include pilots, flight attendants, sailors, officers, engineers, and other personnel who perform various operational tasks and duties on board. Their responsibilities encompass maintaining passenger safety, ensuring operational security, and contributing to the efficient functioning of the transportation mode. | CDC yellow book^8^ |
|  | **Displaced persons**: Someone or groups of individuals who have been forced or obliged to flee or to leave their homes or places of habitual residence, either across an international border or within a State, in particular, because of or to avoid the effects of armed conflict, situations of generalized violence, violations of human rights or natural or human-made disasters. | IOM^10^ |
|  | **Humanitarian aid worker** assists people forced from their homes because of conflict or natural disasters. Assistance begins within hours after a disaster and often continues for years. | CDC yellow book^8^ |
|  | **Immigrant:** Someone who moves into a country other than that of their nationality or usual residence so that the country of destination effectively becomes their new country of usual residence. | IOM^10^ |
|  | **International student**: An individual whose primary goal is acquiring (some form of) higher education in a country where they have no permanent residential status and no local citizenship, regardless of the studying method and may travel for the purpose studying abroad, leisure travel during a gap year, providing health care, or participating in humanitarian activities. | CDC Yellow book^8^ |
|  | **Mass gathering**: A planned or spontaneous event where the number of people attending could strain the planning and response resources of the community or country hosting the event. The Olympic Games, The Hajj, and other major sporting, religious, and cultural events are all examples of mass gatherings. | CDC Yellow book^8^ |
|  | **Medical tourists**: This is an international travel to receive evidence-based medical care. | UNWTO^9^ |
|  | **Migrant:** An umbrella term, not defined under international law, reflecting the common lay understanding of a person who moves away from their place of usual residence, whether within a country or across an international border, temporarily or permanently, and for a variety of reasons. The term includes several well-defined legal categories of people, such as migrant workers; persons whose types of movements are legally defined, such as smuggled migrants; as well as those whose status or means of action are not specifically defined under international law, such as international students. | IOM^10^ |
|  | **Repatriation**: The personal right of a prisoner of war, civil detainee, refugee, or of a civilian to return to his or her country of nationality under specific conditions laid down in various international instruments. | IOM^10^ |
|  | **Refugee:** A person who, owing to a well-founded fear of persecution for reasons of race, religion, nationality, membership of a particular social group or political opinion, is outside the country of his nationality and is unable or, owing to such fear, is unwilling to avail himself of the protection of that country; or who, not having a nationality and being outside the country of his former habitual residence as a result of such events, is unable or, owing to such fear, is unwilling to return to it. | IOM^10^ |
|  | **Sex tourist:** An individual travelling to a different continent to engage in sexual activity, typically with commercial sex workers. It is different from having casual sex during travel with fellow travellers or locals. | CDC yellow book^8^ |
|  | **Sports tourist:** This is the travel experience of the tourist who either observes as a spectator or actively participates in a sporting event generally involving commercial and non-commercial activities of a competitive nature. | UNWTO^9^ |
|  | **Traveller:** A person who moves between different geographic locations for any purpose and duration. | UNWTO^9^ |
|  | **Tourist:** A person who does not reside in the country of arrival and is admitted to that country temporarily (under tourist visas if required) for purposes of leisure, recreation, holiday, visits to friends or relatives, health or medical treatment, or religious pilgrimage. A tourist must spend at least a night in a collective or private accommodation in the receiving country, and the duration of their stay must be at most 12 months. | IOM^10^ |
|  | **VFR-traveller**: A person who currently resides in a higher-income country and returns to their former home (in a lower-income country) for the purpose of visiting friends and/or relatives. More broadly, family members (e.g., children, partners) born in the VFR traveller’s higher-income country of residence are also included in this traveller category. | CDC yellow book^8^ |
|  | **Visitor:** An individual taking a trip to a main destination outside their usual environment, for less than a year, for any main purpose (business, leisure, or other personal purpose) other than to be employed by a resident entity in the country or place visited. | UNWTO^9^ |
|  | **Undocumented migrant:** A non-national who enters or stays in a country without the appropriate documentation. | IOM^10^ |
| **Duration of trip** | |  |
|  | **Diaspora: Someone who is a migrant** or descendants of migrants whose identity and sense of belonging, either real or symbolic, have been shaped by their migration experience and background. They maintain links with their homelands and each other based on a shared sense of history, identity, or mutual understanding in the destination country. | IOM^10^ |
|  | **Long-term traveller**: Someone who contemplates an extended stay in a specific region exceeding six months. | CDC yellow book^8^ |
|  | **Short-term migrant:** A person who changes his or her place of usual residence for more than three months but less than a year (12 months). Except in cases where the movement to that country is for purposes of recreation, holiday, visits to friends or relatives, business, or medical treatment. | IOM^10^ |
|  | **Relocation:** Is an internal humanitarian evacuation and are understood as large-scale movements of civilians, who face an immediate threat to life in a conflict setting, to locations within the same country where they can be more effectively protected. | IOM^10^ |

**Note**: CDC: Centers for Disease Control and Prevention; IOM: International Organization for Migration; UNDESA: United Nations Department of Economic and Social Affairs; UNWTO: United nations world tourism organization.

**S2.** Flow diagram of the article’s eligibility assessment.


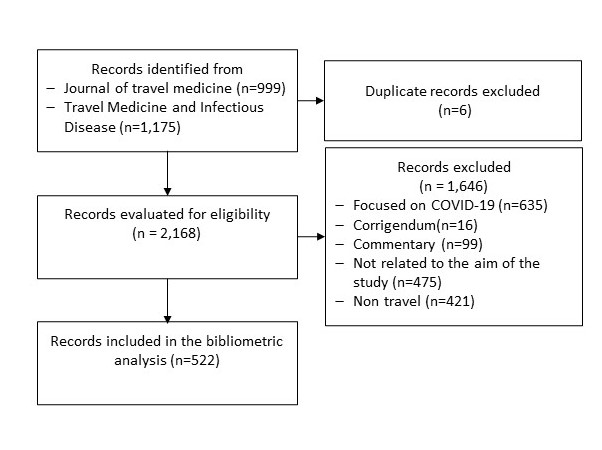

Supplement: Supplementary_material_taae048 [file supplementary_material_taae048.docx]
